# Supplementary material for: Bean Sprouts, Lettuce, and Milk as Water Sources in Tenebrio molitor Larval Growth
Source: Animals (Basel). 2024 Mar 14;14(6):895. doi: 10.3390/ani14060895 (PMC10967327; doi:10.3390/ani14060895)
Supplement: Supplementary file 1 [file animals-14-00895-s001.zip › animals-2887187-supplementary.docx]

Bean sprouts, lettuce, and milk as water sources in *Tenebrio molitor* larval growth

Seokhyun Lee ^1^, Andrew Wange Bugenyi ^2,3^, Hakkyo Lee ^1,2,4^ and Jaeyoung Heo ^1,2,4^ *

^1^ Department of Animal Biotechnology, Jeonbuk National University, Jeonju-si 54896, Republic of Korea

^2^ Department of Agricultural Convergence Technology, Jeonbuk National University, Jeonju-si 54896,

Republic of Korea

^3^ National Agricultural Research Organization, Mbarara P.O. Box 389, Uganda

^4^ International Agricultural Development and Cooperation Center, Jeonbuk National University, Jeonju-si 54896, Republic of Korea

***** Correspondence: [jyheo@jbnu.ac.kr](mailto:jyheo@jbnu.ac.kr)

**Supplementary information**

**Supplementary methods**

**Comparison of larval growth under different moisture source treatments; water, milk, and lettuce**

*Larvae and experimental design*

This preliminary experiment was designed to compare the effect of milk, water and lettuce on larval growth when provided alone. For this, a total of nine (9) rearing trays distributed to the three groups (n = 3) were required. Each larval tray had approximately 5000 freshly hatched larvae. These freshly hatched larvae were obtained by placing, approximately 2,000 adult *Tenebrio molitor* beetles in an oviposition tray which was in turn placed in the plastic larval rearing tray (60 × 40 × 15 cm). Each tray was filled with about 1 cm of wheat bran as feed. This arrangement was maintained for three days to allow for egg laying within the rearing trays. At the end of the 3 days, the adult beetles were separated from the larval trays and 2kg of wheat bran added to the rearing trays. During oviposition and throughout larval growth, the facility was maintained under controlled conditions (Temperature = 25 °C, Relative humidity = 60%, and a 12-hour light–dark cycle). The trays were stacked 15 cm above each other to allow for aeration.

*Moisture treatments*

The *T. molitor* eggs hatched into larvae after about 10 days after oviposition. The moisture treatments were then initiated 20 – 30 days post hatching. Starting with 7g of the moisture source, the amounts were increased by 15 g in subsequent servings (2 times a week) until 60 to 70 days post hatching when the water quantities per serving were maintained at 140g. The larvae were monitored until 92 days post hatching when the experiment was terminated.

*Larval weights*

We assessed larval weight gain under these moisture treatments by randomly sampling and weighing 100 larvae from each tray using an electronic balance (FX-300 electronic balance; A&D company Ltd, Tokyo, Japan). The measurements were taken at three time points during larval growth; at 64-, 71- and 92-days post hatching.

*Analysis*

A one-way analysis of variance (ANOVA) was used to analyze the water source effect for larval weight. Differences between groups were then determined in a post hoc analysis using the Duncan’s multiple range and were considered significant when *p*-value ≤0.05.

**Supplementary results**

**Supplementary Tables**

**Table S1:** Average larval weight of groups treated with lettuce, milk, or water as a sole water source.

|  | Mean larval weight (mg) | | |
| --- | --- | --- | --- |
| **Treatment group** | **Day 64** | **Day 71** | **Day 92** |
| Lettuce | 23.67±2.08^a^ | 35.00±3.61^a^ | 105.33±2.31^a^ |
| Milk | 22.33±0.58^a^ | 31.00±1.00^a^ | 94.00±2.00^b^ |
| Water | 18.33±0.58^b^ | 24.67±1.15^b^ | 61.33±2.31^c^ |
| ***p*-value** | 0.0056 | 0.0040 | <0.0001 |

Data represent mean ± SD (n=3). Mean larval weights with different superscripts at a point in time, are significantly different according to Duncan’s multiple range test at a cut-off of 0.05

**Table S2**: Average larval weights measured at multiple time points during larval development

|  |  |  |  | Mean larval weight (mg) | | | | | | | | | |
| --- | --- | --- | --- | --- | --- | --- | --- | --- | --- | --- | --- | --- | --- |
| **Treatment group** | **water source** | **Milk inclusion** | **Level** | **Day 10** | **Day 21** | **Day 32** | **Day 46** | **Day 56** | **Day 67** | **Day 77** | **Day 87** | **Day 97** | **Day 108** |
| L-1 | Lettuce | - | Low | 1.00 | 1.00 | 5.33 | 13.00^b^ | 20.67^d^ | 40.67^d^ | 53.33^g^ | 74.67^g^ | 99.33^f^ | 127.67^f^ |
| LM-1 |  | + |  | 1.00 | 1.67 | 4.67 | 13.33^b^ | 28.00^c^ | 50.67^bc^ | 74.67^e^ | 102.00^e^ | 129.33^d^ | 148.67^d^ |
| B-1 | Bean sprout | - |  | 1.00 | 1.33 | 5.00 | 12.67^b^ | 25.67^c^ | 38.67^d^ | 60.00^f^ | 82.00^f^ | 109.33^e^ | 139.33^e^ |
| BM-1 |  | + |  | 1.00 | 1.67 | 4.67 | 13.00^b^ | 30.67^b^ | 48.67^c^ | 71.33^e^ | 101.33^e^ | 130.00^d^ | 149.33^d^ |
| L-2 | Lettuce | - | High | 1.00 | 1.67 | 5.00 | 15.33^a^ | 26.00^c^ | 57.33^b^ | 88.67^d^ | 122.00^d^ | 152.00^c^ | 185.00^c^ |
| LM-2 |  | + |  | 1.00 | 2.00 | 4.67 | 15.33^a^ | 37.33^a^ | 86.67^a^ | 122.00^b^ | 170.00^b^ | 210.00^b^ | 214.00^b^ |
| B-2 | Bean sprout | - |  | 1.00 | 1.67 | 5.33 | 15.00^a^ | 37.33^a^ | 84.67^a^ | 116.00^c^ | 156.67^c^ | 202.00^b^ | 232.00^a^ |
| BM-2 |  | + |  | 1.00 | 2.00 | 5.00 | 14.67^a^ | 37.67^a^ | 82.67^a^ | 134.67^a^ | 189.33^a^ | 232.67^a^ | 237.33^a^ |

B-1, bean sprouts-fed larvae; L-2, double the amount of L-1; B-2, double the amount of B-1; LM-1, 50% lettuce + 50% milk; BM-1, 50% bean sprouts + 50% milk; LM-2, double the amount of LM-1; BM-2, double the amount of BM-1.

Mean larval weights with different superscripts at a point in time, are significantly different according to Duncan’s multiple range test at a cut-off of 0.05.

The following tables (Table S2 – Table S11) present results from the three-way ANOVA tests done at the various stages during development

**Table S3**: Summary table of 3-way ANOVA results for comparisons at Day 10 post hatching

| **Source** | **DF** | **Sum of Squares** | **Mean Square** | **F Value** | ***p*-value** |
| --- | --- | --- | --- | --- | --- |
| level | 1 | 0.000 | 0.000 | . | . |
| water_source | 1 | 0.000 | 0.000 | . | . |
| level*water_source | 1 | 0.000 | 0.000 | . | . |
| milk | 1 | 0.000 | 0.000 | . | . |
| level*milk | 1 | 0.000 | 0.000 | . | . |
| water_source*milk | 1 | 0.000 | 0.000 | . | . |
| level*water_source*milk | 1 | 0.000 | 0.000 | . | . |
| Model | 7 | 0.000 | 0.000 | . | . |
| Error | 16 | 0.000 | 0.000 |  |  |
| Corrected Total | 23 | 0.000 |  |  |  |

R-Square = 0 |Coeff Var = 0 |Root MSE = 0 |Mean = 1

**Table S4**: Summary table of 3-way ANOVA results for comparisons at Day 21 post hatching

| **Source** | **DF** | **Sum of Squares** | **Mean Square** | **F Value** | ***p*-value** |
| --- | --- | --- | --- | --- | --- |
| level | 1 | 1.042 | 1.042 | 5.000 | 0.0399 |
| water_source | 1 | 0.042 | 0.042 | 0.200 | 0.6607 |
| level*water_source | 1 | 0.042 | 0.042 | 0.200 | 0.6607 |
| milk | 1 | 1.042 | 1.042 | 5.000 | 0.0399 |
| level*milk | 1 | 0.042 | 0.042 | 0.200 | 0.6607 |
| water_source*milk | 1 | 0.042 | 0.042 | 0.200 | 0.6607 |
| level*water_source*milk | 1 | 0.042 | 0.042 | 0.200 | 0.6607 |
| Model | 7 | 2.292 | 0.327 | 1.570 | 0.214 |
| Error | 16 | 3.333 | 0.208 |  |  |
| Corrected Total | 23 | 5.625 |  |  |  |

R-Square = 0.407407 |Coeff Var = 28.08834 | Root MSE = 0.456435 | W21 Mean = 1.625

**Table S5**: Summary table of 3-way ANOVA results for comparisons at Day 32 post hatching

| **Source** | **DF** | **Sum of Squares** | **Mean Square** | **F Value** | ***p*-value** |
| --- | --- | --- | --- | --- | --- |
| level | 1 | 0.042 | 0.042 | 0.040 | 0.8375 |
| water_source | 1 | 0.042 | 0.042 | 0.040 | 0.8375 |
| level*water_source | 1 | 0.375 | 0.375 | 0.390 | 0.5404 |
| milk | 1 | 1.042 | 1.042 | 1.090 | 0.3126 |
| level*milk | 1 | 0.042 | 0.042 | 0.040 | 0.8375 |
| water_source*milk | 1 | 0.042 | 0.042 | 0.040 | 0.8375 |
| level*water_source*milk | 1 | 0.042 | 0.042 | 0.040 | 0.8375 |
| Model | 7 | 1.625 | 0.232 | 0.240 | 0.9676 |
| Error | 16 | 15.333 | 0.958 |  |  |
| Corrected Total | 23 | 16.958 |  |  |  |

R-Square = 0.095823 | Coeff Var = 19.74343 | Root MSE = 0.978945 | W32 Mean = 4.958333

**Table S6:** Summary table of 3-way ANOVA results for comparisons at Day 46 post hatching

| **Source** | **DF** | **Sum of Squares** | **Mean Square** | **F Value** | ***p*-value** |
| --- | --- | --- | --- | --- | --- |
| level | 1 | 26.042 | 26.042 | 78.120 | <0.0001 |
| water_source | 1 | 1.042 | 1.042 | 3.120 | 0.0962 |
| level*water_source | 1 | 0.042 | 0.042 | 0.120 | 0.7283 |
| milk | 1 | 0.042 | 0.042 | 0.120 | 0.7283 |
| level*milk | 1 | 0.375 | 0.375 | 1.130 | 0.3046 |
| water_source*milk | 1 | 0.042 | 0.042 | 0.120 | 0.7283 |
| level*water_source*milk | 1 | 0.042 | 0.042 | 0.120 | 0.7283 |
| Model | 7 | 27.625 | 3.946 | 11.840 | <0.0001 |
| Error | 16 | 5.333 | 0.333 |  |  |
| Corrected Total | 23 | 32.958 |  |  |  |

R-Square = 0.83818 | Coeff Var = 4.111693 | Root MSE = 0.57735 | W46 Mean = 14.04167

**Table S7:** Summary table of 3-way ANOVA results for comparisons at Day 56 post hatching

| **Source** | **DF** | **Sum of Squares** | **Mean Square** | **F Value** | ***p*-value** |
| --- | --- | --- | --- | --- | --- |
| level | 1 | 416.667 | 416.667 | 185.190 | <0.0001 |
| water_source | 1 | 140.167 | 140.167 | 62.300 | <0.0001 |
| level*water_source | 1 | 6.000 | 6.000 | 2.670 | 0.1220 |
| milk | 1 | 216.000 | 216.000 | 96.000 | <0.0001 |
| level*milk | 1 | 0.167 | 0.167 | 0.070 | 0.7890 |
| water_source*milk | 1 | 66.667 | 66.667 | 29.630 | <0.0001 |
| level*water_source*milk | 1 | 28.167 | 28.167 | 12.520 | 0.0027 |
| Model | 7 | 873.833 | 124.833 | 55.480 | <0.0001 |
| Error | 16 | 36.000 | 2.250 |  |  |
| Corrected Total | 23 | 909.833 |  |  |  |

R-Square = 0.960432 | Coeff Var = 4.931507 | Root MSE = 1.5 | W56 Mean = 30.41667

**Table S8:** Summary table of 3-way ANOVA results for comparisons at Day 67 post hatching

| **Source** | **DF** | **Sum of Squares** | **Mean Square** | **F Value** | ***p*-value** |
| --- | --- | --- | --- | --- | --- |
| level | 1 | 6,600.167 | 6600.167 | 416.850 | <0.0001 |
| water_source | 1 | 140.167 | 140.167 | 8.850 | 0.0089 |
| level*water_source | 1 | 280.167 | 280.167 | 17.690 | 0.0007 |
| milk | 1 | 840.167 | 840.167 | 53.060 | <0.0001 |
| level*milk | 1 | 20.167 | 20.167 | 1.270 | 0.2757 |
| water_source*milk | 1 | 368.167 | 368.167 | 23.250 | 0.0002 |
| level*water_source*milk | 1 | 368.167 | 368.167 | 23.250 | 0.0002 |
| Model | 7 | 8,617.167 | 1231.024 | 77.750 | <0.0001 |
| Error | 16 | 253.333 | 15.833 |  |  |
| Corrected Total | 23 | 8,870.500 |  |  |  |

R-Square = 0.971441 | Coeff Var = 6.49651 | Root MSE = 3.979112 | W67 Mean = 61.25

**Table S9:** Summary table of 3-way ANOVA results for comparisons at Day 77 post hatching

| **Source** | **DF** | **Sum of Squares** | **Mean Square** | **F Value** | ***p*-value** |
| --- | --- | --- | --- | --- | --- |
| level | 1 | 15,301.500 | 15301.500 | 1412.450 | <0.0001 |
| water_source | 1 | 704.167 | 704.167 | 65.000 | <0.0001 |
| level*water_source | 1 | 504.167 | 504.167 | 46.540 | <0.0001 |
| milk | 1 | 2,688.167 | 2688.167 | 248.140 | <0.0001 |
| level*milk | 1 | 140.167 | 140.167 | 12.940 | 0.0024 |
| water_source*milk | 1 | 228.167 | 228.167 | 21.060 | 0.0003 |
| level*water_source*milk | 1 | 8.167 | 8.167 | 0.750 | 0.3981 |
| Model | 7 | 19,574.500 | 2796.357 | 258.130 | <0.0001 |
| Error | 16 | 173.333 | 10.833 |  |  |
| Corrected Total | 23 | 19,747.833 |  |  |  |

R-Square = 0.991223 | Coeff Var 3.653731 | Root MSE = 3.291403 | W77 Mean = 90.08333

**Table S10:** Summary table of 3-way ANOVA results for comparisons at Day 87 post hatching

| **Source** | **DF** | **Sum of Squares** | **Mean Square** | **F Value** | ***p*-value** |
| --- | --- | --- | --- | --- | --- |
| level | 1 | 28,981.500 | 28981.500 | 5609.320 | <0.0001 |
| water_source | 1 | 1,380.167 | 1380.167 | 267.130 | <0.0001 |
| level*water_source | 1 | 840.167 | 840.167 | 162.610 | <0.0001 |
| milk | 1 | 6,080.167 | 6080.167 | 1176.810 | <0.0001 |
| level*milk | 1 | 433.500 | 433.500 | 83.900 | <0.0001 |
| water_source*milk | 1 | 204.167 | 204.167 | 39.520 | <0.0001 |
| level*water_source*milk | 1 | 20.167 | 20.167 | 3.900 | 0.0657 |
| Model | 7 | 37,939.833 | 5419.976 | 1049.030 | <0.0001 |
| Error | 16 | 82.667 | 5.167 |  |  |
| Corrected Total | 23 | 38,022.500 |  |  |  |

R-Square = 0.997826 | Coeff Var = 1.822068 | Root MSE = 2.27303 | W87 Mean = 124.75

**Table S11:** Summary table of 3-way ANOVA results for comparisons at Day 97 post hatching

| **Source** | **DF** | **Sum of Squares** | **Mean Square** | **F Value** | ***p*-value** |
| --- | --- | --- | --- | --- | --- |
| level | 1 | 40,508.167 | 40508.167 | 1609.600 | <0.0001 |
| water_source | 1 | 2,604.167 | 2604.167 | 103.480 | <0.0001 |
| level*water_source | 1 | 1,441.500 | 1441.500 | 57.280 | <0.0001 |
| milk | 1 | 7,280.167 | 7280.167 | 289.280 | <0.0001 |
| level*milk | 1 | 541.500 | 541.500 | 21.520 | 0.0003 |
| water_source*milk | 1 | 504.167 | 504.167 | 20.030 | 0.0004 |
| level*water_source*milk | 1 | 121.500 | 121.500 | 4.830 | 0.0431 |
| Model | 7 | 53,001.167 | 7571.595 | 300.860 | <0.0001 |
| Error | 16 | 402.667 | 25.167 |  |  |
| Corrected Total | 23 | 53,403.833 |  |  |  |

R-Square = 0.99246 | Coeff Var = 3.173414 | Root MSE = 5.016639 | W97 Mean = 158.0833

**Table S12:** Summary table of 3-way ANOVA results for comparisons at Day 108 post hatching

| **Source** | **DF** | **Sum of Squares** | **Mean Square** | **F Value** | ***p*-value** |
| --- | --- | --- | --- | --- | --- |
| level | 1 | 34,504.167 | 34504.167 | 1217.790 | <0.0001 |
| water_source | 1 | 2,562.667 | 2562.667 | 90.450 | <0.0001 |
| level*water_source | 1 | 1,261.500 | 1261.500 | 44.520 | <0.0001 |
| milk | 1 | 1,600.667 | 1600.667 | 56.490 | <0.0001 |
| level*milk | 1 | 4.167 | 4.167 | 0.150 | 0.7064 |
| water_source*milk | 1 | 450.667 | 450.667 | 15.910 | 0.0011 |
| level*water_source*milk | 1 | 60.167 | 60.167 | 2.120 | 0.1644 |
| Model | 7 | 40,444.000 | 5777.714 | 203.920 | <0.0001 |
| Error | 16 | 453.333 | 28.333 |  |  |
| Corrected Total | 23 | 40,897.333 |  |  |  |

R-Square = 0.988915 |Coeff Var = 2.970925 |Root MSE = 5.322906 | Mean = 179.1667

**Table S13:** ANOVA results for comparisons in crude protein, crude fat, crude ash, gross energy, calcium, and potassium content of harvested larvae

| **Crude Protein (%)** | | | | | |
| --- | --- | --- | --- | --- | --- |
| **Source** | **DF** | **Sum of Squares** | **Mean Square** | **F Value** | ***p*-value** |
| Model | 2 | 0.6619 | 0.3310 | 0.11 | 0.8949 |
| Error | 15 | 44.3702 | 2.9580 |  |  |
| Corrected Total | 17 | 45.0321 |  |  |  |
| R-Square = 0.014698\|Coeff Var = 3.123375 \|Root MSE = 1.719887\| Mean = 55.065 | | | | | |
| **Crude Fat (%)** | | | | | |
| **Source** | **DF** | **Sum of Squares** | **Mean Square** | **F Value** | ***p*-value** |
| Model | 2 | 1.0955 | 0.5478 | 0.19 | 0.8256 |
| Error | 15 | 42.3348 | 2.8223 |  |  |
| Corrected Total | 17 | 43.4303 |  |  |  |
| R-Square = 0.025225\|Coeff Var = 4.978033 \|Root MSE = 1.679976 \| Mean = 33.74778 | | | | | |
| **Crude Ash (%)** | | | | | |
| **Source** | **DF** | **Sum of Squares** | **Mean Square** | **F Value** | ***p*-value** |
| Model | 2 | 0.0920 | 0.0460 | 0.35 | 0.7121 |
| Error | 15 | 1.9868 | 0.1325 |  |  |
| Corrected Total | 17 | 2.0788 |  |  |  |
| R-Square = 0.044262 \|Coeff Var = 8.517569 \|Root MSE = 0.363937\| Mean = 4.272778 | | | | | |
| **Gross Energy (kcal/kg)** | | | | | |
| **Source** | **DF** | **Sum of Squares** | **Mean Square** | **F Value** | ***p*-value** |
| Model | 2 | 57987.9667 | 28993.9833 | 3.5 | 0.0567 |
| Error | 15 | 124370.9399 | 8291.3960 |  |  |
| Corrected Total | 17 | 182358.9066 |  |  |  |
| R-Square = 0.317988\|Coeff Var = 1.350472 \|Root MSE = 91.0571\| Mean = 6742.611 | | | | | |
| **Ca (%)** | | | | | |
| **Source** | **DF** | **Sum of Squares** | **Mean Square** | **F Value** | ***p*-value** |
| Model | 2 | 0.0010 | 0.0005 | 0.22 | 0.805 |
| Error | 15 | 0.0352 | 0.0023 |  |  |
| Corrected Total | 17 | 0.0363 |  |  |  |
| R-Square = 0.028506\|Coeff Var = 29.36598\|Root MSE = 0.048454\| Mean = 0.165 | | | | | |
| **P (%)** | | | | | |
| **Source** | **DF** | **Sum of Squares** | **Mean Square** | **F Value** | ***p*-value** |
| Model | 2 | 0.0129 | 0.0065 | 1.18 | 0.3331 |
| Error | 15 | 0.0819 | 0.0055 |  |  |
| Corrected Total | 17 | 0.0949 |  |  |  |
| R-Square = 0.136356\|Coeff Var = 9.10464\|Root MSE = 0.073899\| Mean = 0.811667 | | | | | |

**Table S14**: ANOVA results for comparisons in essential amino acid content of harvested larvae

| **Arginine (%)** | | | | | |
| --- | --- | --- | --- | --- | --- |
| **Source** | **DF** | **Sum of Squares** | **Mean Square** | **F Value** | ***p*-value** |
| Model | 2 | 0.0090 | 0.0045 | 0.32 | 0.7336 |
| Error | 15 | 0.2142 | 0.0143 |  |  |
| Corrected Total | 17 | 0.2232 |  |  |  |
| R-Square = 0.040472\|Coeff Var = 4.403796\|Root MSE = 0.11949\| Mean = 2.713333 | | | | | |
| **Histidine (%)** | | | | | |
| **Source** | **DF** | **Sum of Squares** | **Mean Square** | **F Value** | ***p*-value** |
| Model | 2 | 0.0007 | 0.0004 | 0.05 | 0.9526 |
| Error | 15 | 0.1146 | 0.0076 |  |  |
| Corrected Total | 17 | 0.1154 |  |  |  |
| R-Square = 0.006453\|Coeff Var = 5.133579\|Root MSE = 0.087413\| Mean = 1.702778 | | | | | |
| **Isoleucine (%)** | | | | | |
| **Source** | **DF** | **Sum of Squares** | **Mean Square** | **F Value** | ***p*-value** |
| Model | 2 | 0.0175 | 0.0088 | 0.75 | 0.4879 |
| Error | 15 | 0.1747 | 0.0116 |  |  |
| Corrected Total | 17 | 0.1922 |  |  |  |
| R-Square = 0.091261\|Coeff Var = 4.581498\|Root MSE = 0.10792\| Mean = 2.355556 | | | | | |
| **Leucine (%)** | | | | | |
| **Source** | **DF** | **Sum of Squares** | **Mean Square** | **F Value** | ***p*-value** |
| Model | 2 | 0.0316 | 0.0158 | 0.57 | 0.5761 |
| Error | 15 | 0.4146 | 0.0276 |  |  |
| Corrected Total | 17 | 0.4463 |  |  |  |
| R-Square = 0.070887\|Coeff Var = 4.339004\|Root = MSE 0.166256\| Mean = 3.831667 | | | | | |
| **Lysine (%)** | | | | | |
| **Source** | **DF** | **Sum of Squares** | **Mean Square** | **F Value** | ***p*-value** |
| Model | 2 | 0.1294 | 0.0647 | 2.17 | 0.1489 |
| Error | 15 | 0.4477 | 0.0298 |  |  |
| Corrected Total | 17 | 0.5771 |  |  |  |
| R-Square = 0.22424\|Coeff Var = 5.576962\|Root MSE = 0.172762\| Mean = 3.097778 | | | | | |
| **Methionine (%)** | | | | | |
| **Source** | **DF** | **Sum of Squares** | **Mean Square** | **F Value** | ***p*-value** |
| Model | 2 | 0.0090 | 0.0045 | 1.53 | 0.2477 |
| Error | 15 | 0.0442 | 0.0029 |  |  |
| Corrected Total | 17 | 0.0532 |  |  |  |
| R-Square = 0.169799\|Coeff Var = 7.108655\|Root MSE = 0.054263\| Mean = 0.763333 | | | | | |
| **Phenylalanine (%)** | | | | | |
| **Source** | **DF** | **Sum of Squares** | **Mean Square** | **F Value** | ***p*-value** |
| Model | 2 | 0.0225 | 0.0113 | 2.42 | 0.1229 |
| Error | 15 | 0.0699 | 0.0047 |  |  |
| Corrected Total | 17 | 0.0924 |  |  |  |
| R-Square = 0.243867\|Coeff Var = 3.722613\|Root MSE = 0.068248\| Mean = 1.833333 | | | | | |
| **Threonine (%)** | | | | | |
| **Source** | **DF** | **Sum of Squares** | **Mean Square** | **F Value** | ***p*-value** |
| Model | 2 | 0.0012 | 0.0006 | 0.10 | 0.9065 |
| Error | 15 | 0.0945 | 0.0063 |  |  |
| Corrected Total | 17 | 0.0957 |  |  |  |
| R-Square = 0.013002\|Coeff Var = 4.193933\|Root MSE = 0.079359\| Mean = 1.892222 | | | | | |
| **Valine (%)** | | | | | |
| **Source** | **DF** | **Sum of Squares** | **Mean Square** | **F Value** | ***p*-value** |
| Model | 2 | 0.0048 | 0.0024 | 0.09 | 0.9150 |
| Error | 15 | 0.4038 | 0.0269 |  |  |
| Corrected Total | 17 | 0.4086 |  |  |  |
| R-Square = 0.011775\|Coeff Var = 4.610045\|Root MSE = 0.164066\| Mean = 3.558889 | | | | | |

**Table S15**: ANOVA results for comparisons in non-essential amino acid content of harvested larvae

| **Alanine (%)** | | | | | |
| --- | --- | --- | --- | --- | --- |
| **Source** | **DF** | **Sum of Squares** | **Mean Square** | **F Value** | ***p*-value** |
| Model | 2 | 0.0152 | 0.0076 | 0.11 | 0.8960 |
| Error | 15 | 1.0310 | 0.0687 |  |  |
| Corrected Total | 17 | 1.0462 |  |  |  |
| R-Square = 0.014539\|Coeff Var = 5.89893\|Root MSE = 0.262175\| Mean = 4.444444 | | | | | |
| **Aspartic acid (%)** | | | | | |
| **Source** | **DF** | **Sum of Squares** | **Mean Square** | **F Value** | ***p*-value** |
| Model | 2 | 0.0157 | 0.0079 | 0.2 | 0.8240 |
| Error | 15 | 0.6004 | 0.0400 |  |  |
| Corrected Total | 17 | 0.6161 |  |  |  |
| R-Square = 0.025485\|Coeff Var = 5.058365\|Root MSE = 0.200058\| Mean = 3.955 | | | | | |
| **Cystine (%)** | | | | | |
| **Source** | **DF** | **Sum of Squares** | **Mean Square** | **F Value** | ***p*-value** |
| Model | 2 | 0.0081 | 0.00405 | 1.14 | 0.3448 |
| Error | 15 | 0.0531 | 0.00354 |  |  |
| Corrected Total | 17 | 0.0612 |  |  |  |
| R-Square = 0.132353\|Coeff Var = 10.02774\|Root MSE = 0.059498\| Mean = 0.593333 | | | | | |
| **Glutamic acid (%)** | | | | | |
| **Source** | **DF** | **Sum of Squares** | **Mean Square** | **F Value** | ***p*-value** |
| Model | 2 | 0.1219 | 0.0610 | 1.04 | 0.3767 |
| Error | 15 | 0.8770 | 0.0585 |  |  |
| Corrected Total | 17 | 0.9990 |  |  |  |
| R-Square = 0.122069\|Coeff Var = 4.365558\|Root MSE = 0.241803\| Mean = 5.538889 | | | | | |
| **Glycine (%)** | | | | | |
| **Source** | **DF** | **Sum of Squares** | **Mean Square** | **F Value** | ***p*-value** |
| Model | 2 | 0.0097 | 0.0048 | 0.26 | 0.7738 |
| Error | 15 | 0.2783 | 0.0186 |  |  |
| Corrected Total | 17 | 0.2880 |  |  |  |
| R-Square = 0.033606\|Coeff Var = 4.794273\|Root MSE = 0.136211\| Mean = 2.841111 | | | | | |
| **Proline (%)** | | | | | |
| **Source** | **DF** | **Sum of Squares** | **Mean Square** | **F Value** | ***p*-value** |
| Model | 2 | 0.1683 | 0.0842 | 2.52 | 0.1140 |
| Error | 15 | 0.5013 | 0.0334 |  |  |
| Corrected Total | 17 | 0.6696 |  |  |  |
| R-Square = 0.251394\|Coeff Var = 5.368034\|Root MSE = 0.182811\| Mean = 3.405556 | | | | | |
| **Serine (%)** | | | | | |
| **Source** | **DF** | **Sum of Squares** | **Mean Square** | **F Value** | ***p*-value** |
| Model | 2 | 0.0124 | 0.0062 | 0.79 | 0.4718 |
| Error | 15 | 0.1180 | 0.0079 |  |  |
| Corrected Total | 17 | 0.1305 |  |  |  |
| R-Square = 0.095311\|Coeff Var = 4.584005\|Root MSE = 0.0887\| Mean = 1.935 | | | | | |
| **Tyrosine (%)** | | | | | |
| **Source** | **DF** | **Sum of Squares** | **Mean Square** | **F Value** | ***p*-value** |
| Model | 2 | 0.0371 | 0.0186 | 0.30 | 0.7418 |
| Error | 15 | 0.9144 | 0.0610 |  |  |
| Corrected Total | 17 | 0.9515 |  |  |  |
| R-Square = 0.039038\|Coeff Var = 8.122999\|Root MSE = 0.246894\| Mean = 3.039444 | | | | | |
